# Forest Bathing Always Makes Sense: Blood Pressure-Lowering and Immune System-Balancing Effects in Late Spring and Winter in Central Europe

**DOI:** 10.3390/ijerph18042067

**Published:** 2021-02-20

**Authors:** Agnes Peterfalvi, Matyas Meggyes, Lilla Makszin, Nelli Farkas, Eva Miko, Attila Miseta, Laszlo Szereday

**Affiliations:** 1 Department of Laboratory Medicine, Medical School, University of Pécs, Ifjusag utja 13, 7624 Pécs, Hungary; miseta.attila@pte.hu; 2 Department of Medical Microbiology and Immunology, Medical School, University of Pécs, Szigeti ut 12, 7624 Pécs, Hungary; meggyes.matyas@pte.hu (M.M.); miko.eva@pte.hu (E.M.); szereday.laszlo@pte.hu (L.S.); 3 Institute of Bioanalysis, Medical School, University of Pécs, Szigeti ut 12, 7624 Pécs, Hungary; lilla.makszin@aok.pte.hu (L.M.); nelli.farkas@aok.pte.hu (N.F.)

**Keywords:** forest bathing, forest walking, season, spring, winter, blood pressure, NK cells, CD69, TIM-3, immune system

## Abstract

Various formats of forest bathing have been receiving increasing attention owing to their perspectives in health promotion and the treatment of chronic lifestyle diseases. The majority of field studies are still being conducted in the Far Eastern region, and they often make psychological assessments mainly in the green season. In our pretest–posttest field experiment, twelve healthy, working-age volunteers participated in a 2-h leisurely forest walking program, first in the green season (May) and then in the winter season (January), in the Mecsek Hills, next to Pécs, Hungary. Systolic blood pressure decreased after the trips both in late spring and in the winter. Based on changes in the expressions of CD69, an early activation marker, NKG2D, a major recognition receptor, perforin, granzyme B, and TIM-3, an inhibitory immune checkpoint molecule, on CD8+ cytotoxic T, NK, NK^dim^, NK^bright^, and NKT cells, we detected the stimulation of NK^bright^ cells and activation of all examined immune cell subsets in the green season. In the winter, a slight activating and an interesting balancing effect regarding TIM-3 could be observed considering our finding that basal (pretest) TIM-3 expression by NK cells was significantly lower in the winter. Our work expands the knowledge on and potentials of forest medicine.

## 1. Introduction

The effects of the forest environment on human health and well-being have become a growing field of research interest in the last decade due to its overall beneficial impact. Field experiments were initialized in Japan investigating the effects of a forest bathing trip (“Shinrin-yoku”), which is traditionally a three-day/two-night trip to forest areas involving short, leisurely walks within the forest [1]. Li and colleagues found in a series of studies that the natural killer (NK) cell number and activity and the percentages of perforin-, granulysin-, and granzyme A/B-expressing cells among peripheral blood lymphocytes significantly increased after a forest bathing trip compared to before the trip in healthy male [2] as well as healthy female subjects [3]. A similar city trip to places without forests did not lead to such immunological changes [4]. A forest bathing trip also decreased the adrenaline concentration in urine [3,4], while a city trip did not [4].

The scope of the research field gradually expanded following these principal studies of Li et al. regarding the examined parameters, the characteristics of the forest environment, the duration of exposure to these environments, and the participants. The majority of the field experiments, however, were still conducted in Japan or other Far Eastern countries, such as Korea, Taiwan, or China.

A one-day trip to a forest park involving two 2-h walking sessions—a morning and an afternoon—also resulted in the enhancement of NK activity, number, and expression of cytolytic molecules, as well as enhancing the drop in blood cortisol and urinary adrenaline concentrations [5]. In a similarly conducted study, blood pressure (BP) and urinary dopamine and noradrenaline levels significantly decreased [6]. After a two-day (one night) forest therapy program with 2.5- and 1-h forest walks on the first day and then handcrafting on the second day, systolic BP was found decreased in middle-aged women [7]. A significant reduction in BP was observed in another study, too, involving office workers, who took part in a 1-day forest therapy program with more than 4 h of various activities at the forest site [8]. Significantly lower pulse rates and salivary cortisol levels were revealed in middle-aged females following approximately 3 h of strolling, deep breathing, and resting in the forest on a forest therapy day [9]. The pulse rate reduced significantly also in middle-aged males during two 80-min walking sessions in a forest park, one in the morning and another one in the afternoon, compared to urban walking [10]. A one-day forest recreation program, conducted by Bielinis and co-workers in Poland, including altogether more than 4 h of forest viewing, listening to sounds, touching forest items, cuddling up to a tree, and short walking repeated three times consecutively at three nearby locations led to a significantly lower systolic BP, mean arterial pressure, and pulse rate [11].

A single 2-h exposure to a forest environment reduced serum interleukin-8 and tumor necrosis factor-alpha and raised glutathione peroxidase levels compared to results measured in an urban environment among university students, suggesting an inflammation-reducing and antioxidant effect [12]. A significant decrease was recorded in both the systolic and diastolic BP of working-age people who participated in a 2-h forest bathing, which involved slowly walking around the forest [13]. Yu et al. also observed a significantly lower systolic and diastolic BP and pulse rate in middle-aged and elderly participants after a 2-h forest bathing program including guided stimulation of visual, auditory, olfactory, and tactile senses while walking around the forest site [14].

Even shorter visits to different forest sites or forest parks yielded detectable physiological changes. Both a 15-min viewing of the forest landscape and a 15-min walk in the forest setting were found to lower the BP, pulse rate, and salivary cortisol concentration in healthy male university students [15,16]. In another study, the pulse rate and salivary cortisol level reduced markedly following viewing the forest site for 15 min [17]. An approximately 15-min forest walk decreased the heart rate (HR) in young men [18], as well as in young women [19]. In a study conducted in Helsinki, Finland, a 15-min period of viewing a large urban forest in a sitting position was associated with a lower systolic BP and lower HR, and the subsequent 30-min walking period was associated with a lower HR [20].

A three-day bamboo forest therapy session, including staying at, viewing, and walking around the experimental sites, also decreased the BP and HR and increased NK cell activity and the levels of perforin, granulysin, and granzyme A/B, while it decreased the level of corticosterone in peripheral blood lymphocytes of young male participants [21]. The percentage of activating NK cells rose after a five-day/four-night forest trip with altogether seven 1.5-h walking sessions compared to before the trip [22]; furthermore, the percentage of NK cells was higher in subjects living in a forest environment than in those living in an urban environment [22].

Various formats of forest bathing have been demonstrated to have a favorable impact on hypertension or high-normal BP [23,24,25,26,27,28], chronic heart failure [29,30,31], and chronic obstructive pulmonary disease in elderly patients [32], and on metabolic syndrome [33], chronic widespread pain [34], and breast cancer, when applied as adjuvant forest therapy [35].

Further examinations of the physiological effects of forest environments, as well as their psychological and mental impacts, have been well synthesized in detail in some reviews [36,37,38,39,40,41,42,43].

Considering the small number of field experiments conducted outside the Far Eastern region and investigating immunological effects, the primary aim of our study was to observe the immunological changes regarding the number and function of CD8+ T cells and NK and NKT cells, and also the cardiovascular effects in working-age people, generated by a single session of a 2-h forest bathing in the nearby woods next to Pécs, Hungary, the location of our university.

The following immune cells or subsets and expressed molecules were in the scope of our investigations.

CD8+ cytotoxic T cells, which are components of the adaptive or acquired immune system, and NK cells, which belong to the innate or natural immune system, are both effector lymphocytes recognizing and killing virus-infected or tumor cells [44,45,46]. One pathway of the cytolytic process involves the release of the pore-forming molecule perforin and proteolytic enzymes such as the granzymes from cytotoxic granules inducing apoptosis and thus elimination of the target cells [44,45,46,47,48]. During granule exocytosis, the lysosomal-associated membrane protein-1 (LAMP-1 or CD107a) presents on the effector cell membrane. CD107a is upregulated on the surface of cytotoxic CD8+ T cells [49] and NK cells [50] following stimulation-induced degranulation, and CD107a expression correlates with NK cell-mediated lysis of target cells, such as K562 cells, rendering CD107a a marker of NK cell functional activity [50,51].

Natural killer group 2 member D (NKG2D) is a major recognition receptor for the detection and elimination of various target cells, expressed both on NK cells and CD8+ T cells [52]. In CD8+ T cells, the role of NKG2D is costimulatory, whereby it enhances T cell receptor (TCR) activation and T cell function [53], while in NK cells, NKG2D is an activating receptor able to mediate direct killing of target cells [46,53]. NK cells can be divided into two major subsets based on the density of CD56 on the cell surface. CD56^dim^ NK cells bear low-density expression of CD56, whereas CD56^bright^ NK cells express CD56 at a high density [46,54]. Natural killer T (NKT) cells are lymphocytes originally defined as cells co-expressing T cell (a specific, semi-invariant TCR) and NK cell markers (CD3+CD56+ cells) [55]. NKT cells are involved in the response to infections and also in antitumor responses, and intensive research is undertaken related to them in the field of cancer immunotherapy. NKT cells have several subsets characterized by unique molecular markers and various functions [56,57].

CD69 appears rapidly on the surface of the plasma membrane following activation of several immune cells including T cells and NK cells. Therefore, CD69 is considered to be an early marker of leukocyte activation [58,59]. Immune checkpoint molecules are inhibitory receptors on the surface of various immune cells, especially on T cells, negatively regulating the immune response to prevent autoimmune reactions to self-antigens. Besides autoimmunity, such receptors play a role in the immune defense against infectious diseases and also in antitumor immunity [60]. Immune checkpoint molecules include programmed death-1 (PD-1) [61,62] and T cell immunoglobulin and mucin protein-3 (TIM-3) [63], among others, which are overexpressed on exhausted T cells having progressively lost their effector functions [61,62,63].

## 2. Materials and Methods

### 2.1. Participants

Twelve working-age volunteers living and working in the town of Pécs, Hungary, or its agglomeration were recruited to participate in the study. All subjects were active workers during the accomplishment of the complete study with ages ranging from 25 to 63 years. Demographic data are provided in Table 1. Due to the relatively broad range of ages representing working people, strict medical restrictions were used to exclude unhealthy or potentially unhealthy individuals from the experiments. Autoimmune diseases, malignancy, acute or chronic inflammation, infection, thyroid disorders, hypertension, cardiovascular, pulmonary, liver, or kidney diseases, liver or kidney function tests more than 20% higher than the upper limit of normal in the examining clinical laboratory, diabetes, neuromusculoskeletal problems, pregnancy, abuse of alcohol or substances, and prior or current mental illnesses were all exclusion criteria. One person out of all participants was a regular smoker. Moreover, regular walking, excursion, or exercise in green spaces were also exclusion criteria; furthermore, no forest walking, excursion, or exercise was allowed in the last 4 weeks before the experiment based on the findings of Li et al. [4], who found that the effects of forest bathing on NK cells may last for even 30 days. All participants were provided with a detailed written description of the study protocol and the planned sample collections and measurements, as well as the exclusion criteria, and were enrolled in the study only after giving their full consent. The experimental procedure was officially approved by the Regional Research Ethics Committee of the Medical Centre, Pécs, Hungary (ethical registration number: 8099-PTE). The study protocol conforms to the ethical guidelines of the 2013 revised version of the Declaration of Helsinki.

### 2.2. Study Site and Design

**Figure 1:**
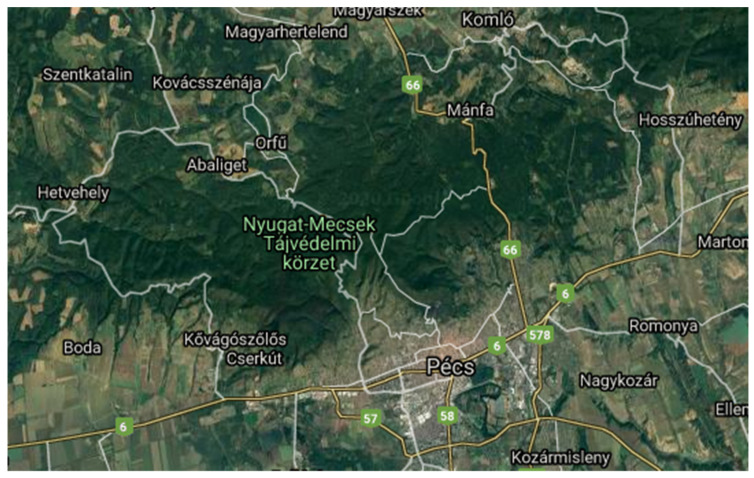
The study site in the Mecsek medium mountains, north of Pécs, the location of the University of Pécs (source: Google Maps).

In this pretest–posttest field experimental study, the participants walked for 2 h at an easy pace with short rests in the nearby recreational woodland area next to the town of Pécs. Pécs is located south of the slopes of the western part of the Mecsek medium mountains (Figure 1). The woodland area surrounding Pécs is a recreational territory with pleasant, sign-posted routes. The start, as well as the endpoint, of the experimental walking route was the so-called tv tower in the hills at 535 m altitude, which is easily accessible by car within 20 min from the town and has a parking lot as well.

**Figure 2:**
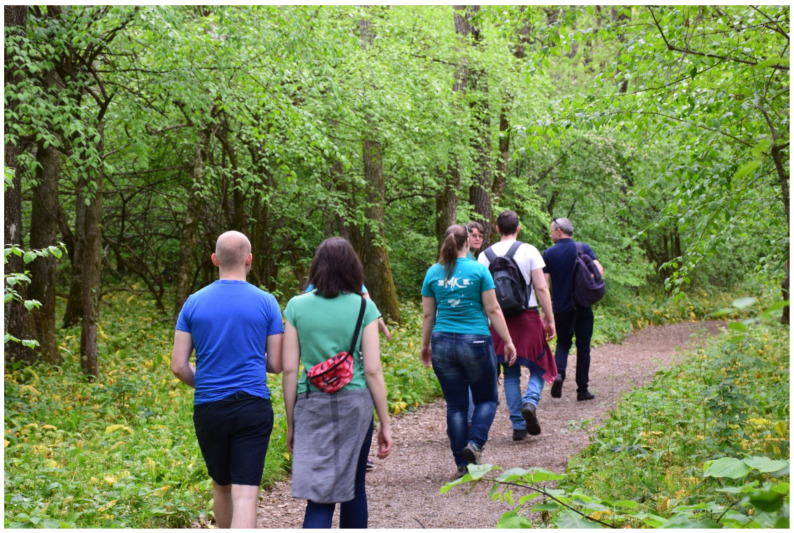
Participants walking on the tourist route in May.

The experimental forest walk was 6.3 km long, carried out in 2 h along sign-posted tourist routes including two viewpoints with the sight of the western part of Pécs and the Mecsek Hills, and also including the southern side of the so-called Rotary Promenade, a local classical walking trail (Figure 2). The elevation during the whole walking route was 76 m. The woodland area where the walk was going through is a so-called hilltop forest integrating northern and southern environmental effects leading to the development of a special ecological habitat. Overall, an oak forest with sub-Mediterranean features is located in this territory, grown on rocky (chalk) and shallow ground. The area is covered mainly by various oak species such as *Quercus pubescens*, *Quercus cerris*, and *Quercus virgiliana*, as well as by ash (*Fraxinus ornus*) and linden (*Tilia tomentosa*). Additional tree species include *Sorbus torminalis* and *Acer campestre*. The mean age of the trees is almost 115 years, and no logging or active management is carried out in this region. The ground is covered with diverse vegetation including several species of shrubs and herbaceous plants. At the time of the May trip, fading wild garlic (*Allium ursinum*) was also present in abundance along the pathway in some areas.

In order to enhance immersion into the forest environment in the recreational woodland site, the walk was slow and five short resting periods were also integrated: two at the 2 viewpoints, watching the scenery, then one for quietly listening to the sounds of the forest, one for carefully viewing remote and close items in the forest, and one for touching at least one optional plant, tree, or rock. Participants were encouraged to breathe deeply and slowly throughout the walk and to take notice of the scents of the forest as well. The experimental walk was conducted by one of the researchers. The group kept a comfortable pace for everyone between the resting periods, and no subject indicated any sign of fatigue or feeling tired.

The first 2-h forest walking session took place in May 2017, followed by another one in January 2018 performed by the same participants. In May, the temperature was 23 °C and the relative humidity was 47% at the time of the walk, on an overall sunny day with no perceptible wind. In January, the temperature was 4 °C and the relative humidity was 81% also on a sunny day with no perceptible wind, but the landscape was covered with 12 cm snow.

Three days prior to the day of the experimental forest walking program, in the morning of a normal working day, between 8 and 9 a.m., participants visited the laboratory in our university, and venous blood was drawn from a cubital vein by a phlebotomist. The next day after the forest walk, another venous blood sample was obtained from the participants in the morning, between 8 and 9 a.m., in order to avoid any possible effect of the circadian rhythm on the investigated parameters.

On the day of the experimental forest walk, the 12 participants and two researchers met at 2 p.m. at the starting point of the walk, in the parking lot, where they arrived individually or in small groups (2–3 persons together) by car. After gathering and sitting for 10 min at rest, blood pressure and pulse rate were measured with a portable automatic blood pressure monitor (OMRON M3 Intellisense, Omron Corporation, Kyoto, Japan). After completing the walk, blood pressure and pulse rate were again measured in the same way following a 10-min rest in a sitting position.

### 2.3. Measurements

Venous blood samples were used to measure routine laboratory parameters: complete blood count and differential cell counts (Sysmex XN 2000, Sysmex, Japan), ions, liver enzymes, urea, creatinine, C-reactive protein, thyroid-stimulating hormone, and fasting plasma glucose (Cobas 6000, Roche, Switzerland), and to perform immunological measurements.

Peripheral blood mononuclear cells (PBMC) were separated by centrifugation (1800 rpm, 20 min, 20 °C) on a density gradient (Ficoll-Paque, GE Healthcare, Uppsala, Sweden). Separated cells were counted and stored at −80 °C in a mechanical freezer after washing in RPMI 1640 medium (Lonza, Verviers, Belgium) and resuspending in human serum (Biowest, Nuaillé, France) containing 10% DMSO (Sigma-Aldrich, Le Chesne, France) for cryoprotection. On the day of the flow cytometric analysis, cells were thawed and washed twice in RPMI 1640 medium (Lonza) to remove DMSO.

For the fluorescent labeling of PBMC, the following monoclonal antibodies were used: fluorescein isothiocyanate (FITC)-conjugated mouse anti-human CD3 (Clone: HIT3α, BD Biosciences, San Jose, CA, USA), Brilliant Violet (BV) 510-conjugated mouse anti-human CD8 (Clone: SK1, BD Biosciences), allophycocyanin (APC)-conjugated mouse anti-human CD56 (Clone: B159, BD Biosciences), phycoerythrin (PE)-cyanine (Cy)7-conjugated mouse anti-human NKG2D (Clone: 1D11, BD Biosciences), BV421-conjugated mouse anti-human CD69 (Clone: FN50, BD Biosciences), PE-conjugated anti-human PD-1 (Clone: PD1.3, Beckman-Coulter, Marseille, France), APC/Cy7-conjugated anti-human TIM-3 (Clone: F38-2E2, BioLegend, San Diego, CA, USA), peridinin chlorophyll protein (PerCP)-Cy5.5-conjugated mouse anti-human perforin (Clone: δG9, BD Biosciences), Pacific Blue-conjugated anti-human granzyme B (Clone: GB11, BioLegend).

Cell surface staining was performed with 10^6^ thawed PMBC/sample in 100 µL phosphate-buffered saline (PBS, PAA Laboratories, Pasching, Austria)/tube using the above listed fluorochrome-labeled monoclonal antibodies to CD3, CD8, CD56, NKG2D, CD69, PD-1, and TIM-3. After incubation for 30 min at room temperature in the dark, samples were centrifuged after adding 2 mL PBS (2000 rpm, 5 min, 20 °C), and the cells were resuspended in 300 µL PBS containing 1% paraformaldehyde (PFA). Tubes were stored at 4 °C in the dark during the preparation of the intracellular staining. After surface labeling with antibodies to CD3, CD8, and CD56, cells were fixed in 4% PFA, permeabilized with 1:10 diluted FACS Permeabilizing Solution (BD Biosciences, San Jose, CA, USA), labeled with monoclonal antibodies to perforin and granzyme B (listed above), and finally fixed with PBS containing 1% PFA.

The cytotoxic potential of the examined cell types was assessed by the flow cytometric analysis of CD107a surface expression in a functional test. PBMCs were thawed and washed, then the number of viable cells was determined by trypan blue (Sigma-Aldrich, Gillingham, UK) dye exclusion. Then, 10^6^ viable cells were incubated for 4 h at 37 °C in 1 mL RPMI 1640 medium containing 10% fetal bovine serum (Gibco, Paisley, UK), and 1 µg/mL ionomycin (Sigma-Aldrich) and 25 ng/mL phorbol myristate acetate (PMA, Sigma-Aldrich) for cell stimulation in the presence of PE-conjugated mouse anti-human CD107a antibodies (BD BioSciences). After washing in PBS, surface labeling with antibodies to CD3, CD8, and CD56 was performed, and, finally, the cells were fixed with 1% PFA.

All labeled samples were analyzed with a BD FACS Canto II flow cytometer (BD Biosciences) with BD FACS Diva V6 software (BD Biosciences) for data acquisition. Flow cytometer settings were checked using Cytometer Setup and Tracking beads (BD Biosciences) according to the manufacturer’s protocol. To determine compensation values, spectral overlap values were measured for all fluorophores, via single-color controls. Analysis of the flow cytometric data was performed with FCS Express V4 (De Novo Software, Pasadena, CA, USA).

All 4 samples were collected from each single participant of the study, so samples before and after the late spring trip and samples before and after the winter walk of the same subject were thawed and processed, labeled, and analyzed parallelly, on the same day, by the same person, under identical conditions.

The examined cells, their identifying cell surface markers, and the analyzed molecules expressed on the surface or produced intracellularly are summarized in Table 2.

### 2.4. Statistics

Statistical analysis was performed using the SPSS version 26.0 statistics software (IBM Corporation, Armonk, NY, USA). A normality test was used to determine whether sample data were drawn from a normally distributed population. Depending on normality, comparisons of the means and ranks of the different seasons’ pre–post parameters were achieved by the paired samples t-test and the Wilcoxon test, respectively, in order to determine statistically significant parameters (*p* < 0.05). The independent samples t-test and the Mann–Whitney test were used for the comparison of the seasons’ pre (basal level) parameters. The *p*-values less than 0.05 were considered to be significant.

## 3. Results

**Figure 3:**
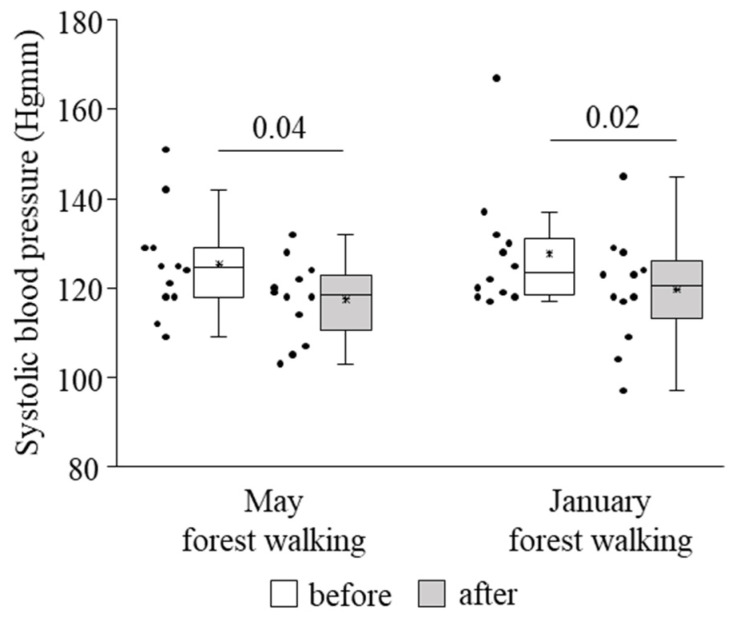
Systolic blood pressure prior to and following the late spring (May) and winter (January) forest walks. The middle line within the box represents the median, the middle dot within the box represents the mean, the boxes indicate the interquartile ranges between the 25th and 75th percentiles, and the whiskers show the most extreme observations in the box plot. Individual dots left from the boxes represent individual data values. Differences were considered statistically significant for *p*-values < 0.05. Statistically significant *p*-values are indicated on the horizontal lines outside the boxes.

Systolic blood pressure was found to become significantly lower owing to the forest trip in late spring as well as in winter (Figure 3). No effect could be detected on diastolic blood pressure or pulse rate (data not shown).

Values of routine clinical laboratory parameters, including total white blood cell count, granulocyte, lymphocyte, and monocyte counts, red blood cell count, hemoglobin concentration, platelet count, serum sodium and potassium concentrations, serum levels of aspartate aminotransferase (ASAT), alanine aminotransferase (ALAT), urea, creatinine, and C-reactive protein, and fasting plasma glucose, measured before the trip did not differ from the values registered after the trip, either in May or in January (data not shown).

Regarding immune cell subsets, the proportion of NKT cells increased after the May forest walk, and no other change was found in any subsets, in either walking session (Table 3).

**Figure 4:**
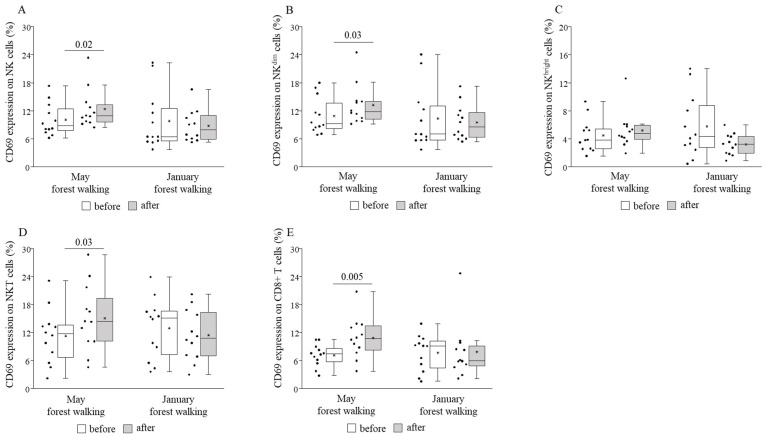
CD69 expressions on total natural killer (NK) (**A**), NK^dim^ (**B**), NK^bright^ (**C**), NKT (**D**), and CD8+ T (**E**) cells prior to and following the late spring (May) and winter (January) forest walks. The middle line within the box represents the median, the middle dot within the box represents the mean, the boxes indicate the interquartile ranges between the 25th and 75th percentiles, and the whiskers show the most extreme observations in the box plot. Individual dots left from the boxes represent individual data values. Differences were considered statistically significant for *p*-values < 0.05. Statistically significant *p*-values are indicated on the horizontal lines outside the boxes.

CD69 expression was shown to become elevated on CD8+ T, total NK, NK^dim^, and NKT cells, while it remained statistically unchanged on NK^bright^ cells following the May intervention, and no alteration could be observed following the January version (Figure 4).

**Figure 5:**
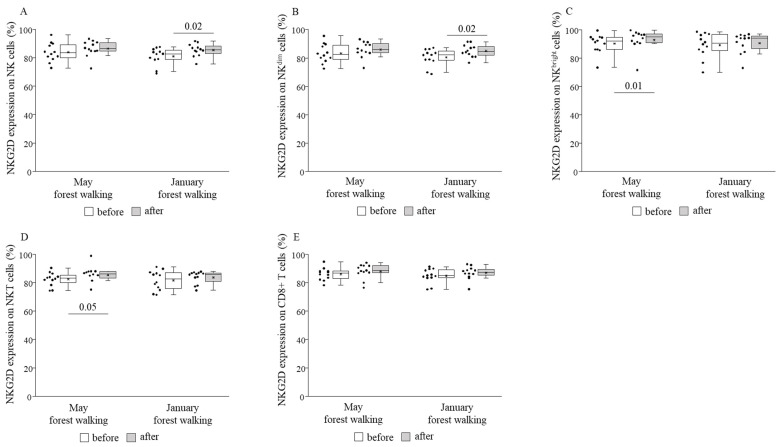
NKG2D expressions on total NK (**A**), NK^dim^ (**B**), NK^bright^ (**C**), NKT (**D**), and CD8+ T (**E**) cells prior to and following the late spring (May) and winter (January) forest walks. The middle line within the box represents the median, the middle dot within the box represents the mean, the boxes indicate the interquartile ranges between the 25th and 75th percentiles, and the whiskers show the most extreme observations in the box plot. Individual dots left from the boxes represent individual data values. Differences were considered statistically significant for *p*-values < 0.05. Statistically significant *p*-values are indicated on the horizontal lines outside the boxes.

NKG2D expression was revealed to be raised on NK^bright^ and NKT cells after the late spring session and on total NK and NK^dim^ cells after the winter session without any change on any other cell subsets in either season (Figure 5).

**Figure 6:**
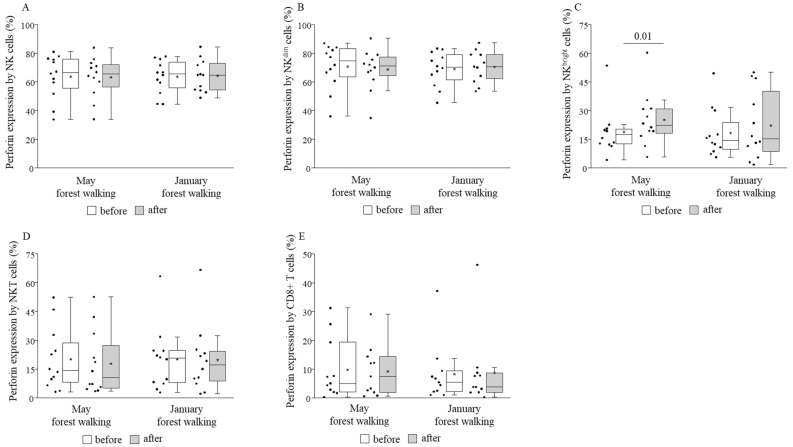
Perforin expressions by total NK (**A**), NK^dim^ (**B**), NK^bright^ (**C**), NKT (**D**), and CD8+ T (**E**) cells prior to and following the late spring (May) and winter (January) forest walks. The middle line within the box represents the median, the middle dot within the box represents the mean, the boxes indicate the interquartile ranges between the 25th and 75th percentiles, and the whiskers show the most extreme observations in the box plot. Individual dots left from the boxes represent individual data values. Differences were considered statistically significant for *p*-values < 0.05. Statistically significant *p*-values are indicated on the horizontal lines outside the boxes.

**Figure 7:**
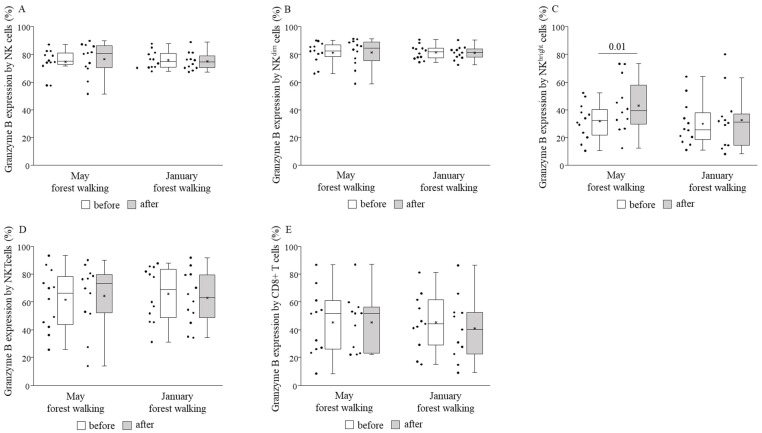
Granzyme B expressions by total NK (**A**), NK^dim^ (**B**), NK^bright^ (**C**), NKT (**D**), and CD8+ T (**E**) cells prior to and following the late spring (May) and winter (January) forest walks. The middle line within the box represents the median, the middle dot within the box represents the mean, the boxes indicate the interquartile ranges between the 25th and 75th percentiles, and the whiskers show the most extreme observations in the box plot. Individual dots left from the boxes represent individual data values. Differences were considered statistically significant for *p*-values < 0.05. Statistically significant *p*-values are indicated on the horizontal lines outside the boxes.

Perforin and granzyme B expressions by NK^bright^ cells were measured to be significantly higher after the forest walk in May, and no other cell subset in either month showed any change regarding such expressions (Figure 6 and Figure 7).

**Figure 8:**
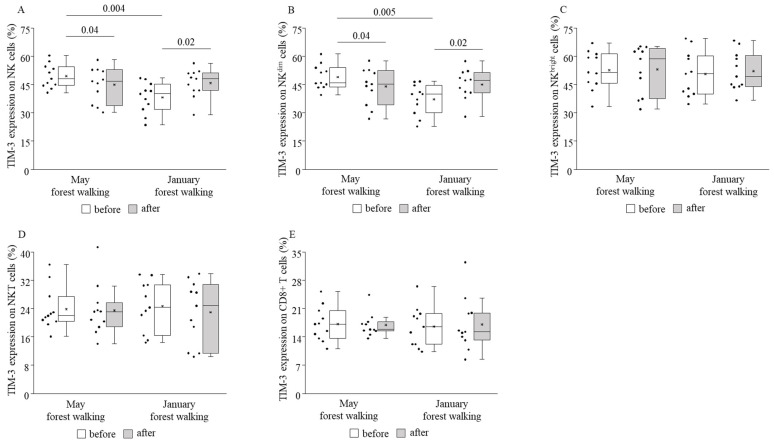
TIM-3 expressions on total NK (**A**), NK^dim^ (**B**), NK^bright^ (**C**), NKT (**D**), and CD8+ T (**E**) cells prior to and following the late spring (May) and winter (January) forest walks. The middle line within the box represents the median, the middle dot within the box represents the mean, the boxes indicate the interquartile ranges between the 25th and 75th percentiles, and the whiskers show the most extreme observations in the box plot. Individual dots left from the boxes represent individual data values. Differences were considered statistically significant for *p*-values < 0.05. Statistically significant *p*-values are indicated on the horizontal lines outside the boxes.

**Figure 9:**
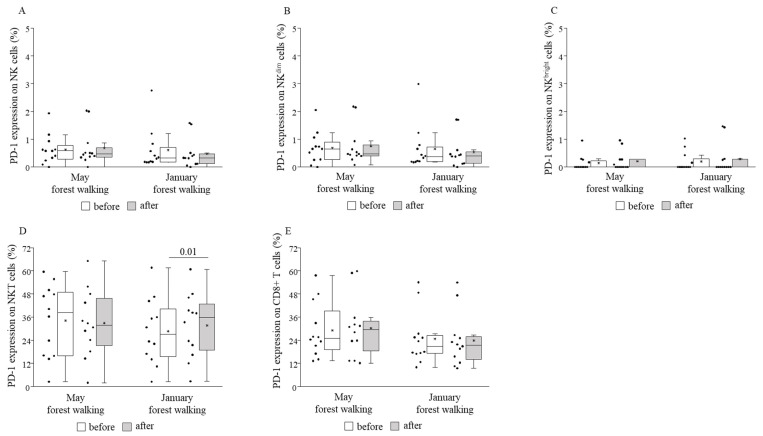
PD-1 expressions on total NK (**A**), NK^dim^ (**B**), NK^bright^ (**C**), NKT (**D**), and CD8+ T (**E**) cells prior to and following the late spring (May) and winter (January) forest walks. The middle line within the box represents the median, the middle dot within the box represents the mean, the boxes indicate the interquartile ranges between the 25th and 75th percentiles, and the whiskers show the most extreme observations in the box plot. Individual dots left from the boxes represent individual data values. Differences were considered statistically significant for *p*-values < 0.05. Statistically significant *p*-values are indicated on the horizontal lines outside the boxes.

TIM-3 expression decreased on total NK and NK^dim^ cells following the forest bathing in May and, on the contrary, increased on total NK and NK^dim^ cells after the same trip in January. No further changes in TIM-3 expressions could be demonstrated when comparing pre and post values (Figure 8). PD-1 expression was raised on NKT cells following the winter tour without any alterations on other cell subsets or in the spring season (Figure 9).

CD107a expressions did not show any significant changes on any cell subsets either in May or in January (data not shown).

When comparing the basal (pretest) values of each examined parameter between late spring and winter, we observed strongly significant differences in the TIM-3 expressions on total NK and NK^dim^ cells, with the winter values being significantly lower than the spring values (Figure 8). May and January values of all the other parameters were similar to each other (Figure 4, Figure 5, Figure 6, Figure 7 and Figure 9).

## 4. Discussion

In our study, twelve actively working adults of various ages participated in a 2-h leisurely forest walking program immersing in the atmosphere of the woods in the Mecsek Hills, next to Pécs, Hungary. The first walk was carried out in late spring, in May, followed by a second identical one in the winter, in January. Certain cardiovascular and immunological parameters were measured before and after each intervention. Systolic blood pressure decreased significantly in both seasons as a consequence of the forest walks. Effects of the May walk included elevated percentages of NKT cells, CD69-expressing total NK, NK^dim^, NKT, and CD8+ cytotoxic T cells, NKG2D receptor-expressing NK^bright^ and NKT cells, and perforin- and granzyme B-expressing NK^bright^ cells, as well as reduced percentages of TIM-3-expressing total NK and NK^dim^ cells. In the winter, in January, the percentages of NKG2D receptor-expressing total NK and NK^dim^ cells were raised, and TIM-3-expressing total NK and NK^dim^ cells, as well as PD-1-expressing NKT cells, also increased. Furthermore, when comparing the basal (pretest) levels of the investigated parameters between late spring and winter, we found that the TIM-3 expression of total NK and NK^dim^ cells was significantly lower in January than in May.

The duration of exposure to the forest environment in field experiments detecting any physiological changes in the subjects varies from 15 min to a couple of days with complex forest bathing programs. Differences in parameters related to the immune response could be demonstrated after a 2-h walk in some studies [1,12] and after longer sessions in others [21,22]. Since our primary aim was to observe potential cellular immunological alterations besides blood pressure and pulse rate monitoring, we did not expect any remarkable changes owing to a forest trip shorter than 2 h. From a preventive medicine perspective, a 2-h leisurely forest walk seems to be feasible even for untrained persons and could regularly be fitted into a healthier lifestyle of actively working people as well. Considering all these aspects, we decided to assess the impact of a 2-h forest trip, both in the green and the leafless season.

The systolic blood pressure-lowering effect demonstrated by our research work is in line with several other studies that revealed similar results following 2-h forest walking [6,13,14]. These studies observed decreased diastolic blood pressure as well, and one experiment also found a reduced pulse rate [14], while one study did not record significant changes in the pulse rate [13]. We could not detect alterations either in diastolic blood pressure or in the pulse rate. There are numerous data available on the generally beneficial impact of various formats of forest bathing on the systolic and/or diastolic component of blood pressure and/or the pulse or heart rate [41,64]. Cardiovascular outcomes connected to nature interventions including forest walking and viewing, though, are highly heterogeneous, according to the evaluation of a recent review [65].

Our study is the first to analyze NK cell subsets, NKT cells, CD8+ cytotoxic T cells, and stimulatory and inhibitory receptors in the framework of forest bathing. Regarding cell percentages within the group of peripheral blood mononuclear cells, we detected no significant changes between before and after the forest trip, except for NKT cells, whose ratio became higher following the trip in late spring. The levels of perforin and granzyme B did not change in total NK and NK^dim^ cells, neither did they change in NKT nor CD8+ T cells, but both showed significant increases in NK^bright^ cells after the late spring walk. Li and co-workers in numerous studies [2,3,4,5] and Lyu and colleagues in a bamboo forest therapy study [21] equally found elevated NK cell percentages and perforin-, granzymes A/B-, and granulysin-expressing cells in peripheral blood lymphocytes (PBL), as well as NK cell (PBL) activity. We could not reveal any significant effect on cytotoxic activities of cell subsets indicated as CD107a expression. We could record, on the other hand, significantly raised expressions of CD69, an early activation marker, on almost all examined populations: NK cells, NK^dim^ cells, NKT, and CD8+ T cells, owing to the late spring walk. The latter findings are in accordance with the results of the study conducted by Tsao et al. [22], who obtained elevated percentages of activating NK cells (CD3^−^/CD56^+^/CD69^+^) after a complex forest trip program, without any change in the proportions of NK cells in peripheral blood.

Further expanding the scope of our research, the expression of NKG2D, an activating receptor, increased on NK^bright^ and NKT cells in late spring, while the expression of TIM-3, an inhibitory receptor, decreased on NK and NK^dim^ cells. The two NK cell subsets, NK^dim^ and NK^bright^ cells, have different receptor expression profiles. CD56^dim^ NK cells have high natural cytotoxicity; in turn, CD56^bright^ NK cells are able to produce large amounts of cytokines following stimulation [46,54]. According to the linear model of human NK cell development, CD56^bright^ NK cells mature into CD56^dim^ cells, which subsequently differentiate into adaptive NK cells in response to viral infection [66,67]; furthermore, beyond their cytokine-producing and regulatory role, CD56^bright^ NK cells may also have potent cytotoxic functions as demonstrated in an ex vivo expansion study [68]. The increased NKG2D receptor expression, and the raised perforin and granzyme B production found only in NK^bright^ cells, but not in the rest of the NK cells, suggest that the size of the effect induced by the 2-h leisurely forest walk in the Mecsek Hills was able to stimulate immature cells, but it seems insufficient to impact the cytolytic molecule production of mature cells. Consistently, we detected the activation of all other cell types including total NK and NK^dim^, NKT, and CD8+ T cells, together with decreased inhibition of NK and NK^dim^ cells, without a change in perforin or granzyme B production by any of them. We suppose that on a hypothesized scale of the immune system-enhancing potential of forest bathing, our field experiment generated positive but initial changes to the immune system of the participants.

While Li and co-workers found significant changes in percentages of NK cells, activity, and cytolytic molecule expression even after a 2-h long forest walk (after the first day of forest bathing) in Japanese forests [1], Lyu and colleagues demonstrated such changes after a three-day bamboo forest therapy session [21]. Jung and colleagues, on the other hand, reported no effect of a three-day/two-night forest therapy program on NK cell activity [69], but Tsao et al. revealed activation of NK cells following a five-day/four-night forest trip [22]. While the 2-h leisurely forest walk generated an increased NK cell number and activity and expression of anti-cancer proteins in a series of field experiments conducted by Li et al. [1], we could not detect such explicit changes following our 2-h forest walking program; however, activation of all examined cell subsets (by increased expressions of CD69 and NKG2D and decreased expression of TIM-3) and increased expressions of perforin and granzyme B by NK^bright^ cells were revealed in late spring. The directions of the changes evoked by forest bathing in the two experimental settings are rather similar, and only the intensity of the effect seems to alter between the two studies. In the works of Li and colleagues, the subjects took part in a 3-day/2-night trip to forest areas and meanwhile stayed at a hotel next to the forest, which was mainly composed of coniferous trees such as Japanese cedar and cypress [1]. Contrary to these conditions, in our experiment, the participants left the town only for 2 h to accomplish the forest walk, and the major tree species were oaks, deciduous trees. Not only the duration of the forest walk itself, but also the duration of the overall exposure to the forest environment may have an impact on the intensity of the evoked effect on the cells of the immune system.

On the other hand, the composition and other features of the forest, which are unique characteristics of the experimental site, may influence, to a great extent, the effects of forest bathing. One study, for example, reported that different tree species, namely, birch, maple, and oak, and different environmental factors have different effects on the blood pressure and heart rate [70]. Due to this, results naturally alter to some extent in similar studies, too. Moreover, the type of the forest at the experimental sites also has a great influence on the forest air, the compounds, and the concentrations of phytoncides present in the air. Various phytoncides from coniferous trees significantly increased human NK activity and the expression of perforin, granulysin, and granzyme A in in vitro experiments [71] and raised the percentages and activity of NK cells as well as the expressions of perforin, granulysin, and granzyme A/B in in vivo human studies [72]. High concentrations of phytoncides were detected in the coniferous forests in the works of Li and colleagues [1], while our study took place in a deciduous forest; moreover, we did not collect and analyze forest air samples, which is a limitation of our experiment. The different composition of the forest air in the two experimental settings may also account for the differences in the findings of our study and that of Li et al. [1].

This is the first study that assessed seasonal variations in the effect of the same forest environment on blood pressure, pulse rate, and immunological parameters in the same subjects. The majority of forest field experiments evaluated the impact of the green season. Some works investigated changes evoked by other seasons: Bielinis and colleagues examined psychological effects (the participants filled in psychological questionnaires) in winter [73,74]; Song and co-workers also examined psychological effects and, moreover, showed a lower heart rate due to short walks in urban parks in fall [75] as well as in winter [76]. A reduction in blood pressure, but not in pulse rate, was observed following a half-hour walk in a deciduous forest without leaves in fall in a field experiment conducted by Janeczko et al. [77]. As for the seasonal impact on the same participants, Bielinis et al. investigated the psychological effects of viewing the landscape for 15 min in spring and winter [78], while Brooks and colleagues reported on three studies dealing with mood measures in different seasons [79]. According to our results, the systolic blood pressure-reducing effect of the 2-h forest walk in late spring could be reproduced in winter, suggesting a persistent beneficial effect of the forest environment on blood pressure. Regarding components of the immune system, the higher expression of the NKG2D-activating receptor on NK and NK^dim^ cells in January also suggests slight immune enhancement, although other activating markers found in late spring were missing in winter.

Interestingly, the expression of the inhibitory receptor PD-1 increased on NKT cells, and, contradictory to the late spring results, the expression of the other examined inhibitory receptor, TIM-3, on NK and NK^dim^ cells also became raised following the winter walk. What is more, the differential basal expression of TIM-3 by NK and NK^dim^ cells in late spring and in winter was an additional and surprising finding of our study, since we expected no notable differences in any immunological parameters in time. We were unable to find any data in the literature relevant to seasonal variations in TIM-3 expression. Seasonality, though, contributes, to a great extent, to the onset and exacerbation of autoimmune diseases such as multiple sclerosis, systemic lupus erythematosus, psoriasis, and rheumatoid arthritis, with winter and early spring being the risk factors potentially due to low vitamin D levels, low melatonin levels, or the increased incidence of certain infectious diseases [80]. TIM-3, as an inhibitory immune checkpoint molecule, may play a role in the pathogenesis of autoimmune diseases [81,82]. Based on our finding of a significantly lower TIM-3 expression in winter by NK and NK^dim^ cells, we may hypothesize that the reduced inhibition might partially mediate the winter exacerbations. In this case, winter forest bathing may balance immune regulation by elevating TIM-3 expressions, by enhancing a favorable inhibition. On the other hand, the decreased inhibition in winter may simply be an adaptive response to ameliorate the immune defense against pathogens common in the winter. Lacking further investigations and objective data, only hypotheses can be set up, which require further research and enlightenment.

The major limitation of our study is the relatively small number of participants. In the few other field experiments assessing immunological parameters at the cellular level, the number of subjects varied between 11 and 13 in case of actively working and/or older people [2,3,4,5,22], and one study recruited 60 participants, who were college students aged between 19 and 24 [21]. The 12 working-age volunteers in our study participated not only in one forest walking session, but also in another one eight months later, thus enabling us to analyze the effects of a late spring and a winter forest bathing on the same participants, which is a strength of our work. Another limitation is the lack of observation of the duration of forest walking effects. Blood was drawn from the subjects only before and the day after the interventions, both in May and in January, which meant admittance to the laboratory and blood collection four times from a person. We applied for ethical approval with this protocol presuming that six or more times would put too much strain on the potential subjects and discourage them from participating.

The lack of an identical experiment featuring walking in a non-forested city area is a further limitation. Such an experiment would have helped confirm the effects of the forest environment itself. We designed our study as a pretest–posttest field experiment carried out in late spring and then repeated in the winter with the same participants. Finally, another limitation is that we provided only the description of the forest site and the weather conditions but collected no forest air samples and made no analysis of its compounds. We are planning to perform such measurements in future studies.

## 5. Conclusions

In this pretest–posttest experimental forest walking study, blood pressure and pulse rate, and some cellular components of the immune system and their stimulatory and inhibitory receptor expression profiles were investigated before and after a 2-h intervention in late spring and winter involving the same subjects.

Our work demonstrates a blood pressure-lowering and an immune function-enhancing effect of forest walking, both in May and in January, with a more pronounced effect in late spring. Our study expands related immunological research, deepens our knowledge on the cellular and molecular changes generated, and contributes to promoting forest bathing as a possible useful and effective tool of preventive medicine and forest therapy. More research in the field, especially physiological changes evoked by winter forests, is warranted.

## Figures and Tables

**Table 1:** Demographic data of the participants.

Demographic Data	
No. of participants	*n* = 12
Females	*n* = 7
Males	*n* = 5
Age (years) (mean, range)	38.5 (25–63)
BMI (mean, range)	24.71 (19.72–30.42)
Smoking	*n* = 1
Status	Active workers

BMI: body mass index.

**Table 2:** Flow cytometric staining of the analyzed cells.

Analyzed Cells	Markers	Surface Staining	Intracellular Staining	Functional Assay
cytotoxic T cells	CD3+ CD8+	NKG2D, CD69, PD-1, TIM-3	perforin, granzyme B	CD107a
NK cells total	CD3- CD56+	NKG2D, CD69, PD-1, TIM-3	perforin, granzyme B	CD107a
NK^dim^ cells	CD56+ dim	NKG2D, CD69, PD-1, TIM-3	perforin, granzyme B	CD107a
NK^bright^ cells	CD56++ bright	NKG2D, CD69, PD-1, TIM-3	perforin, granzyme B	CD107a
NKT cells	CD3+ CD56+	NKG2D, CD69, PD-1, TIM-3	perforin, granzyme B	CD107a

**Table 3:** Proportions of the examined cell subsets (100% = all peripheral blood mononuclear cells; data are presented as mean ± standard deviation); * *p* = 0.045 compared to the before value.

	Late Spring		Winter	
Cell Subset	Before %	After %	Before %	After %
CD8+ T	17.41 (±5.73)	18.12 (±6.53)	17.52 (±6.98)	18.23 (±5.96)
NK total	17.94 (±6.66)	17.77 (±7.42)	21.83 (±8.84)	20.21 (±9.97)
NK^dim^	15.96 (±6.05)	16.17 (±6.94)	20.25 (±8.67)	18.42 (±9.39)
NK^bright^	2.11 (±1.15)	1.75 (±0.84)	1.73 (±1.06)	1.99 (±1.03)
NKT	8.06 (±9.36)	8.91 (±10.48) *	8.30 (±10.39)	7.87 (±8.85)

## Data Availability

The data presented in this study are available on request from the corresponding author. The data are not publicly available as the participants gave permission to learn and handle their personal and research generated data only to researchers involved in this study.
